# Early Right Ventricular Apical Pacing-Induced Gene Expression Alterations Are Associated with Deterioration of Left Ventricular Systolic Function

**DOI:** 10.1155/2017/8405196

**Published:** 2017-08-08

**Authors:** Haiyan Xu, Xiongwei Xie, Jiangjin Li, Yuanyuan Zhang, Changsong Xu, Jing Yang

**Affiliations:** ^1^Department of Cardiology, Huai'an First People's Hospital, Nanjing Medical University, Huai'an, Jiangsu, China; ^2^Department of Medical Laboratory, Huai'an First People's Hospital, Nanjing Medical University, Huai'an, Jiangsu, China; ^3^Department of Medical Ultrasonics, Huai'an First People's Hospital, Nanjing Medical University, Huai'an, Jiangsu, China

## Abstract

The chronic high-dose right ventricular apical (RVA) pacing may have deleterious effects on left ventricular (LV) systolic function. We hypothesized that the expression changes of genes regulating cardiomyocyte energy metabolism and contractility were associated with deterioration of LV function in patients who underwent chronic RVA pacing. Sixty patients with complete atrioventricular block and preserved ejection fraction (EF) who underwent pacemaker implantation were randomly assigned to either RVA pacing (*n* = 30) group or right ventricular outflow tract (RVOT) pacing (*n* = 30) group. The mRNA levels of OPA1 and SERCA2a were significantly lower in the RVA pacing group at 1 month's follow-up (both *p* < 0.001). Early changes in the expression of selected genes OPA1 and SERCA2a were associated with deterioration in global longitudinal strain (GLS) that became apparent months later (*p* = 0.002 and *p* = 0.026, resp.) The altered expressions of genes that regulate cardiomyocyte energy metabolism and contractility measured in the peripheral blood at one month following pacemaker implantation were associated with subsequent deterioration in LV dyssynchrony and function in patients with preserved LVEF, who underwent RVA pacing.

## 1. Introduction

It has proven that long-term right ventricular apical (RVA) pacing may result in ventricular dyssynchrony and heart failure (HF) [1–3]. Subsequently, some approaches have done to eliminate the adverse effects of RVA pacing, such as minimal ventricular pacing (VP), alternative right ventricular (RV) sites, and biventricular (BiV) pacing. However, ventricular pacing cannot be reduced in many patients with atrioventricular (AV) block. The alternative right ventricular pacing sites have not yet been proven to provide clinical benefits in randomized studies. On the other hand, BiV pacing has a relatively higher complication rate and higher cost. RVA is still the commonest pacing site around the world.

Although most patients with preserved ejection fraction (EF) tolerate RVA pacing, new-onset HF has been shown in such patients during long-term follow-up [4, 5]. The underlying mechanism of the detrimental effects of chronic RVA pacing effects on patients with preserved left ventricular (LV) function is not fully understood. It is known that certain genes regulate mitochondrial energy metabolism, excitation-contraction coupling, and contractile proteins are involved in HF development [6, 7]. In particular, the normal contraction of the heart is an energy-dependent process. Optic atrophy 1 (OPA1) plays a critical role in cardiac energetics. Alterations in the expression of sarcoplasmic reticulum calcium ATPase2a (SERCA2a) lead to contractile deficiency and pathological remodeling in HF. Although the aetiology of LV dysfunction is different, electromechanical dyssynchrony contributes to myocardial structural remodeling in HF. In clinical practice, LV ejection fraction (LVEF) measured by conventional echocardiography is the most frequently used parameter in the evaluation of cardiac systolic function. However, it may be somewhat insufficient in detecting early signs of cardiac dysfunction. Three-dimensional (3D) speckle-tracking strain echocardiography (STE), a more accurate and reliable method to determine ventricular myocardial function, can detect subtle myocardial dysfunction [8]. Global longitudinal strain (GLS) is a novel predictor of reduction of LV systolic function in patients with preserved EF for chronic RVA pacing [9]. Therefore, we sought to determine whether RVA pacing-induced early expression changes of genes regulating the cardiac energy metabolism and excitation-contraction coupling proteins measured in the peripheral blood were associated with deterioration of GLS and LVEF that becomes evident in the long term in patients with preserved LV function.

## 2. Materials and Methods

### 2.1. Study Population

This was a prospective randomized controlled study, conducted between January 2012 and December 2015. Patients with complete AV block and preserved LVEF (≥50%) were enrolled. Subjects with heart failure, valvular heart disease, cardiomyopathy, documented coronary artery disease, atrial fibrillation, pulmonary disease, and renal insufficiency were excluded from the study. Blood samples were collected at baseline and 1 month. Serial pacemaker checkups, echocardiography, and clinical assessments were performed at baseline and 1, 6, 12, and 24 months. The investigation protocol was approved by the institutional review board and the ethics committee of Huai'an First People's Hospital and complied with the Declaration of Helsinki. Consent forms were obtained from all study participants.

Patients with complete AV block and preserved LVEF (≥50%) were randomly assigned in 1 : 1 ratio to receive either RVA pacing (*n* = 30) or right ventricular outflow tract (RVOT) (*n* = 30) pacing. Each patient received a dual-chamber rate-modulated (DDDR) pacemaker. The ventricular leads were placed in the right ventricular apex in RVA pacing group, while the ventricular leads were placed the right ventricular outflow tract septum in RVOT pacing group. Implantation of ventricular leads in the RVOT septum was performed under fluoroscopic guidance according to the tip of the electrode directed to the spine 45° to the left anterior oblique view. The RVOT septum was confirmed according to the surface pacemaker electrocardiogram (ECG) which showed a negative or isoelectric vector in lead I and positive Q, R, and S waves in leads II, III, and aVF [10]. The operators were blinded to ultrasonographic data.

### 2.2. Expression of Genes

Fasting serum samples were stored at −80°C. Whole-blood RNA was then isolated as described previously [11]. Total RNA was isolated using TRIzol reagent (Invitrogen, Carlsbad, CA). cDNA syntheses was performed with 1 *μ*g of total RNA according to the instruction of SYBR Premix Ex Taq™ II (Tli RnaseH Plus) (Takara) in 20 *μ*L reactions. Measurements of mRNA levels were analyzed by SYBR PCR Master Mix reagent kits (Takara, Tokyo, Japan). All real-time PCR (RT-PCR) reactions were carried out on the ABI 7900 Fast Real-Time System (Applied Biosystems, CA, USA). A preamplification denaturation was performed at 95°C for 30 sec, followed by real-time PCR with a thermal profile that included 40 cycles of denaturation at 95°C for 5 sec, annealing and extension at 60°C for 30 sec, and melting curve generation from 65 to 95°C. All RT-PCR experiments were repeated at least three times. The glyceraldehyde-3-phosphate dehydrogenase (GAPDH) was used as internal control under the same conditions, and the relative expression of genes was calculated with the 2^−ΔΔCt^ method. Primer sequences for OPA1, SERCA2a, and GAPDH were predesigned and validated by the company. The primer pairs used in RT-PCR analysis were listed in Supplementary Table S1 available online at https://doi.org/10.1155/2017/8405196.

### 2.3. Echocardiography

Standard transthoracic echocardiography was performed using an iE-33 ultrasound system (Philips Medical Systems, N.A., Bothell, WA, USA). Standard techniques were used to obtain two-dimensional (2D), M-mode, and Doppler measurements according to the American Society of Echocardiography guidelines [12]. Real-time three-dimensional (3D) echocardiography was performed on the same ultrasound machine with X3-1, a fully sampled matrix array transducer. Both 2D and 3D echocardiographic images were analyzed using the Qlab 7 software (Philips Medical Systems). We measured GLS, global circumferential strain (GCS), global radial strain (GRS), and standard deviation index of three-dimensional strain (SDI) using the speckle-tracking method. Systolic dyssynchrony was calculated by the use of the standard 16-segment model. End-diastolic volume (EDV), end-systolic volume (ESV), and LV ejection fraction (LVEF) were measured automatically by the software from the single full-volume acquisition in the 3D mode. Data of three consecutive beats were digitally stored to ensure optimal data quality. Baseline and follow-up echocardiographic examinations were performed on the same machine. All echocardiographic examinations for each patient were performed and analyzed by the same experienced echocardiographer (CSX) who was blinded to clinical data and group division.

Intra- and interobserver variability of both strain measurements and LVEF were examined in 10 randomly selected patients, by two investigators (CSX and HYX). The same primary operator analyzed selected data twice at intervals of greater than 2 weeks. Both operators were blinded to the results of the first measurement and from each other. Intra- and interobserver variability was calculated as absolute and relative differences between measurements and presented as coefficient of variation.

### 2.4. Statistical Analysis

Continuous data were expressed as mean ± SD and compared between the RVA pacing group and the RVOT pacing group by nonpaired Student *t*-test. Categorical data were summarized as frequencies and percentages, which were analyzed using the chi-square test or Fisher exact test as appropriate. The comparison of the repeated measurements was carried out using repeated measurement of ANOVA test. Univariable and multivariable linear regression analyses were used to evaluate the association between gene expression alterations and echo variable changes. All tests were two sided where a *p* < 0.05 was considered statistically significant. Statistical analyses were performed with SPSS 18 software (SPSS Inc., Chicago, IL).

## 3. Results

### 3.1. Patient Characteristics

A total of 60 patients were enrolled in this study and were randomly assigned in 1 : 1 ratio to receive either RVA (*n* = 30) or RVOT (*n* = 30) pacing. The patients were recruited in the period of the 30th of January 2012 and 31st of December 2013. No patients were lost during the 2 years of follow-up after implantation. Vital data were available for all patients at the end of follow-up. After a follow-up of 2 years, four patients had new onset of HF in the RVA pacing group; however, only one patient with new-onset HF was observed in the RVOT pacing group. As shown in Table 1, no significant difference was observed regarding clinical parameters, medication use, and echocardiographic parameters at baseline between the two groups. Initial QRS duration was similar between the RVA pacing and RVOT pacing groups. However, the mean paced QRS duration was significantly longer in the RVA pacing group than in the RVOT pacing group (154 ± 12 versus 132 ± 11 ms, *p* < 0.001) at the end of follow-up.

### 3.2. Alterations in the Expression of Genes

**Figure 1:**
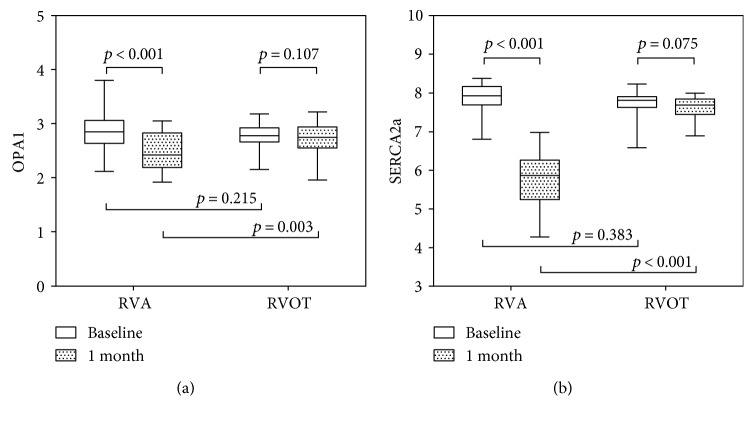
Comparison of changes in mRNA levels OPA1 and SERCA2a between the RVA pacing group and the RVOT pacing group. (a) Changes in mRNA levels of OPA1 at baseline and 1 month after implantation. (b) Changes in mRNA levels of SERCA2a at baseline and 1 month after implantation. RVA, right ventricular apical; RVOT, right ventricular outflow tract.

There was no significant difference in mRNA levels of the genes measured at the baseline between the RVA pacing and RVOT pacing groups (Figure 1). At 1 month, the mRNA levels of OPA1 were significantly lower in the RVA pacing group than in the RVOT pacing group (*p* = 0.003). The mRNA levels of OPA1 decreased significantly in the RVA pacing group compared with initial levels (*p* < 0.001), while that was not observed in the RVOT pacing group (*p* = 0.107). A similar result was observed for SERCA2a between the two groups (*p* < 0.001). Compared with the baseline value, the mRNA levels of SERCA2a decreased in the RVA pacing group at 1 month (*p* < 0.001). In the RVOT pacing group, the difference was not statistically significant (*p* = 0.075) between the initial and final mRNA levels of SERCA2a.

### 3.3. Alterations in Echocardiographic Parameters

To monitor cardiac structure and function, echocardiography was performed for all patients before cardiac pacemaker implantation and during the follow-up. There were no significant differences in any echocardiographic parameters between the RVA pacing group and the RVOT pacing group at baseline (Table 2). At 1 month, there was no difference in LVEF, GLS, GRS, GLS, and SDI between the two groups (Table 2). At 12 months' follow-up, the GLS and SDI were significantly different between the two groups (*p* = 0.044, *p* = 0.001, resp.). At 24 months' follow-up, notable changes were observed between the two groups regarding GLS (*p* = 0.015), SDI (*p* = 0.001), and LVEF (*p* = 0.034). Changes in GRS (*p* = 0.392) and GCS (*p* = 0.078) showed no differences according to pacing site.

Repeated measures of ANOVA showed significant time × group interaction effects on GLS (*p* < 0.001), LVEF (*p* = 0.030), and SDI (*p* < 0.001). In the RVA pacing group, significant time effects were observed for GLS, LVEF, and SDI (*p* < 0.001 for all). Post hoc tests showed a progressive reduction of GLS; the most dramatic changes occurred at 12 months' follow-up (*p* < 0.001) and further deteriorated at 24 months' follow-up (*p* = 0.037). The deleterious effect on SDI showed the same tendency. The absolute reduction of LVEF was 6.2% in the RVA pacing group from baseline to 24 months' follow-up (*p* < 0.001). Significant time × group interaction effects were also observed for GRS and GCS (both *p* < 0.001). Post hoc tests showed GRS was significantly less at 24 months' follow-up than at baseline (*p* < 0.001). Compared to the baseline value, GCS was significantly impaired with RVA pacing at 24 months' follow-up (*p* < 0.001). No significant changes were observed in the RVOT pacing group. The interobserver and intraobserver variabilities for measuring dyssynchrony and LVEF were presented in Supplementary Table S2.

### 3.4. Impaction of Early Gene Expression Alterations to Deterioration in Left Ventricular Systolic Function and Dyssynchrony

Univariable and multivariable linear regression were applied to evaluate for the relationship between alterations in the gene expression and deterioration in LV dyssynchrony and function that became more evident later at the 24 months' follow-up in the RVA pacing group. In univariate linear regression analysis, the altered mRNA levels of genes were not significantly correlated with clinical parameters and the echocardiographic parameters at baseline. A decrease in expression of the gene SERCA2a was significantly associated with a decrease in expression of the gene OPA1 at 1 month's follow-up (*β* = 0.213, 95% CI 0.152–0.274, *p* < 0.001). A decrease in the mRNA levels of OPA1 had a significant association with change in GLS (*β* = −0.066, 95% CI −0.089 to −0.044, *p* < 0.001), LVEF (*β* = 0.017, 95% CI 0.008–0.025, *p* < 0.001), and SDI (*β* = −0.034, 95%CI −0.055 to −0.012, *p* = 0.004) but not GRS and GCS. A decrease in the mRNA levels of SERCA2a was also significantly associated with the change in GLS (*β* = −0.259, 95% CI −0.340 to −0.178, *p* < 0.001), change in LVEF (*β* = 0.074, 95% CI 0.046–0.103, *p* < 0.001), and change in SDI (*β* = −0.138, 95% CI −0.217 to −0.059, *p* = 0.001) (Table 3). The worsening LVEF had a significant association with the deterioration of GLS (*β* = −2.823, 95% CI −3.387 to −2.260, *p* < 0.001) and SDI (*β* = −1.622, 95% CI −2.284 to −0.961, *p* < 0.001). The deterioration of GLS was also associated with the change in SDI (*β* = 0.521, 95% CI 0.316–0.725, *p* < 0.001).

In multivariate linear regression analysis, only delta GLS was independently associated with changes in mRNA levels of OPA1 and SERCA2a (Table 4). The worsening LVEF had a significant association with deterioration of GLS (*β* = −2.507, 95% CI −3.331 to −1.684, *p* < 0.001).

## 4. Discussion

In the present study, we demonstrated that RVA pacing-induced alterations in the expression of genes that regulate cardiomyocyte energy metabolism and contractility one month after implantation were associated with deterioration in LV dyssynchrony and function later in patients with preserved LVEF. In the RVA pacing group, mRNA levels of OPA1 and SERCA2a decreased at 1 month's follow-up, while the dyssynchrony parameters (GLS, GRS, GCS, and SDI) and LVEF had no significant changes. Compared to baseline, the GLS was significantly impaired since 6 months' follow-up. The LVEF, SDI, and GRS were significantly impaired until 12 months' follow-up. Univariate linear regression analysis showed the decreases in mRNA levels of OPA1 and SERCA2a correlated with GLS, LVEF, and SDI. In multivariate linear regression analysis, the changes of GLS were independently associated with decreases in mRNA levels of OPA1 and SERCA2a. The worsening LVEF also had a significant association with deterioration of GLS. In contrast, either the expression of the genes in the peripheral blood or echocardiographic parameters demonstrated statistically significant changes in the RVOT pacing group. Therefore, the changes in OPA1 and SERCA2a gene mRNA levels appeared to be associated with GLS deterioration and LVEF decline in the RVAP pacing group.

For decades, a wealth of literature have been showing that RVA pacing may lead to left ventricular systolic dysfunction and heart failure [1, 2]. It is well known that RVA pacing induces electromechanical dyssynchrony, which leads to adverse hemodynamic effects and myocardial remodeling [13–15]. Clinical studies showed significant detrimental effects of RVA pacing in patients with reduced baseline LVEF [2, 3] and high percentage VP [16]. However, clinically relevant LV dysfunction was observed rather infrequently in patients with preserved LVEF during long-term follow-up [17, 18]. Furthermore, identification of those patients at greatest risk of suffering the negative effects of RVA pacing will optimize therapeutic strategies to prevent HF from developing.

Peripheral blood gene expression profiling has emerged as a powerful tool for investigating the pathophysiology of heart disease [19–21]. Mitochondria provide an essential source of energy for cellular processes, which is particularly important in heart muscle cells. Mitochondrial dynamics—balanced fusion and fission—shapes mitochondria to meet metabolic demands and plays a vital role in cellular physiology and pathology [22, 23]. A fragmentation of the mitochondrial network is observed in HF [24]. OPA1 in the inner membrane is one of the fusion proteins. OPA1 is also a critical regulator of mitochondrial respiration [25]. Chen et al. [26] have described decreased myocardial levels of OPA1 in ischemia-induced heart failure. In a consistent way, overexpression of OPA1 may help ameliorate the cardiac dysfunction in diabetes cardiomyopathy [27]. On the other hand, HF development is associated with the genes that regulate excitation-contraction coupling. The SERCA2a, a pivotal component of calcium handling, plays a crucial role in modulating cardiac contraction and relaxation. Reduced SERCA2a expression and function has been documented in HF [28–30]. There is accumulating evidence that restore SERCA2a expression and that activity can improve mechanical function and electrical stability in HF [31, 32]. Similar to previous study, we observed decreased transcript levels of the OPA1 and SERCA2a in patients with preserved LVEF receiving RVA pacing at 1 month of follow-up. Our data showed that a decrease in mRNA levels of the genes was correlated with a reduction of GLS and a decrease in LVEF at 24 months following RVA pacing. Therefore, alterations in the expression of the genes at an early stage measured in peripheral blood showed potential for identifying patients at high risk of subsequent deterioration in LV systolic function.

We observed that deterioration of GLS had a correlation with LVEF decline, which was consistent with previous reports [33]. LVEF has been the most commonly used indicator of cardiac dysfunction. However, there has been increasing concern that LVEF is insufficiently sensitive to distinguish small changes of LV systolic dysfunction in patients with preserved EF. Latest advances in 3D STE are capable to accurately assess myocardial deformation in all three dimensions simultaneously in a full-volume data set [34, 35]. A number of experimental studies demonstrated a good correlation between 3D STE strain dyssynchrony and 3D LVEF [36], as well as between 3D STE strain dyssynchrony and 3D SDI [37]. The RVA pacing induced LV radial dyssynchrony, LV systolic longitudinal shortening, and LVEF decline [14, 38]. In line with experimental findings by Ahmed et al. [9], this study showed the GLS was evidently reduced at 12 months' follow-up and further deteriorated at the second year in the RVA pacing group. However, the LVEF significantly declined at 24 months' follow-up. The worsening LVEF had a significant association with deterioration of GLS (*p* < 0.001). Our data would suggest that GLS may be more sensitive to monitor change in LV function. GLS was more reliable and reproducible than other parameters. In our study, GLS presented good interobserver and intraobserver agreement. The lack of association of GCS, GRS, and SDI with the change in LVEF might be explained by the lower reproducibility of these parameters.

The present study has several limitations. Firstly, the relatively small sample size limits our findings to some extent, and larger studies are needed to clarify these results. Secondly, measurement of mRNA in peripheral blood is not the gold standard. The relationship between the altered expression of mRNA in peripheral blood mononuclear cells and LV diastolic dysfunction has been reported in patients with preserved LV ejection fraction [39]. Therefore, it is still a reliable index for early noninvasive screening. Lastly, although the LVEF was significantly impaired at 24 months after pacemaker implantation, a longer follow-up time should be warranted to detect the progressive alteration in LV function.

## 5. Conclusions

We demonstrated a significant association between early expression changes of genes regulating cardiomyocyte energy metabolism and contractility and a late decline in GLS and LVEF in patients with preserved LV function, who underwent long-term RVA pacing. Therefore, measurement of mRNA in peripheral blood at one month following RVA pacing shows potential for identifying patients at high risk of subsequent deterioration of LV function before any changes in echocardiographic parameters were detected. Further studies are needed to determine the optimal approach for identifying patients who might benefit from other pacing modalities during a very early phase.

## Supplementary Material

Table S1. Primer sequences used for quantitative real-time PCR. Table S2. Intra- and inter-observer variability of LVEF and strain parameters.

## Figures and Tables

**Table 1:** Summary of patient characteristics.

Variables	Overall	RVA	RVOT	*p* Value
Age	67.9 ± 8.4	67.1 ± 7.5	68.7 ± 9.3	0.477
Male (%)	33 (55.0)	17 (56.7)	16 (53.3)	0.795
BMI	24.1 ± 2.37	23.7 ± 2.5	24.4 ± 2.2	0.294
SBP	139 ± 21	142 ± 22	137 ± 20	0.377
DBP	73 ± 17	75 ± 18	71 ± 16	0.372
Hypertension (%)	28 (46.7)	15 (50)	13 (43.3)	0.605
Diabetes mellitus (%)	12 (20.0)	5 (16.7)	7 (23.3)	0.519
Coronary heart disease (%)	13 (21.7)	6 (20.0)	7 (23.3)	0.754
ACEI/ARB (%)	19 (31.7)	8 (26.7)	11 (36.7)	0.405
Beta-blockers (%)	12 (20.0)	7 (23.3)	5 (16.7)	0.519
Statins (%)	17 (28.3)	8 (26.7)	9 (30)	0.774
Prepacing QRS duration (ms)	103 ± 13	102 ± 11	105 ± 14	0.375
Paced QRS duration (ms)	143 ± 16	154 ± 12	132 ± 11	<0.001
BNP	242.5 (203.3–298.5)	235.4 (198.1–289.5)	259.3 (210.3–309.0)	0.469
LAV (mL)	33.9 ± 4.4	33.4 ± 3.9	34.3 ± 4.9	0.435
LVEDV (mL)	100.9 ± 11.9	103.2 ± 11.4	98.6 ± 12.2	0.139
LVESV (mL)	38.4 ± 6.1	37.8 ± 5.1	39.0 ± 7.0	0.452
LVEF (%)	62.7 ± 5.7	63.0 ± 5.4	62.4 ± 6.1	0.704
EA ratio	1.03 ± 0.21	0.99 ± 0.20	1.07 ± 0.22	0.121
GLS (%)	−13.0 ± 2.8	−13.2 ± 2.4	−12.9 ± 3.0	0.746
GRS (%)	29.0 ± 5.7	29.7 ± 5.7	28.2 ± 5.2	0.311
GCS (%)	−20.3 ± 5.0	−20.8 ± 4.4	−19.8 ± 5.5	0.441
SDI	12.2 ± 2.1	12.1 ± 1.9	12.3 ± 2.1	0.852
MWD (m)	377.9 ± 39.0	381.9 ± 31.1	374.9 ± 44.9	0.562

BMI: body mass index; SBP: systolic blood pressure; DBP: diastolic blood pressure; ACEI: angiotensin-converting enzyme inhibitors; ARB: angiotensin receptor blockers; VP: ventricular pacing; BNP: brain natriuretic peptide; LAV: left atrial volume; LVEDV: left ventricular end-diastolic volume; LVESV: left ventricular end-systolic volume; LVEF: left ventricular ejection fraction; GLS: global longitudinal strain; GRS: global radial strain; GCS: global circumferential strain; SDI: standard deviation index of three-dimensional strain; MWD: 6-min walking distance.

**Table 2:** LVEF and strain parameters in the RVA pacing and RVOT pacing groups during the follow-up.

Variables	RVA	RVOT	*p* Value
*LVEF (%)*			
Baseline	63.0 ± 5.4	62.4 ± 6.1	0.704
1 month	61.8 ± 5.3	61.3 ± 5.2	0.678
6 months	60.5 ± 5.8	61.5 ± 5.4	0.506
12 months	59.4 ± 6.5^∗^	61.1 ± 5.1	0.258
24 months	56.7 ± 7.6^∗^^#^	60.4 ± 5.3	0.034
*GLS (%)*			
Baseline	−13.1 ± 2.5	−12.9 ± 3.0	0.746
1 month	−12.9 ± 2.7	−12.5 ± 3.0	0.639
6 months	−12.7 ± 2.8^∗^	−12.4 ± 3.4	0.510
12 months	−10.6 ± 2.8^∗^^#^	−12.3 ± 3.6	0.044
24 months	−9.9 ± 3.0^∗^^#^	−12.1 ± 3.7	0.015
*GRS (%)*			
Baseline	29.7 ± 5.7	28.2 ± 5.2	0.311
1 month	29.4 ± 6.2	28.1 ± 5.3	0.385
6 months	28.4 ± 6.4	28.1 ± 5.4	0.547
12 months	27.9 ± 6.5^∗^^#^	27.8 ± 5.5	0.966
24 months	25.9 ± 6.6^∗^^#^	27.3 ± 6.3	0.392
*GCS (%)*			
Baseline	−20.8 ± 4.4	−19.8 ± 5.5	0.441
1 month	−20.5 ± 4.6	−19.7 ± 5.6	0.502
6 months	−20.1 ± 5.1	−19.4 ± 5.9	0.622
12 months	−19.2 ± 5.4^∗^^#^	−19.0 ± 6.0	0.875
24 months	−15.7 ± 5.6^∗^^#^	−18.6 ± 6.6	0.078
*SDI (%)*			
Baseline	12.2 ± 2.0	12.3 ± 2.1	0.852
1 month	12.8 ± 2.4	12.7 ± 2.5	0.876
6 months	13.1 ± 2.3	13.0 ± 2.6	0.959
12 months	15.5 ± 2.4^∗^^#^	13.1 ± 2.7	0.001
24 months	16.4 ± 3.5^∗^^#^	13.5 ± 2.7	0.001

LVEF: ejection fraction left ventricular; RVA: right ventricular apical; RVOT: right ventricular outflow tract; GLS: global longitudinal strain; GRS: global radial strain; GCS: global circumferential strain; SDI: standard deviation index of three-dimensional strain. ^∗^*p* < 0.05 versus baseline in the same group; ^#^*p* < 0.05 versus 1 month in the same group.

**Table 3:** Multivariate linear regression analysis for the changes in mRNA levels of OPA1 and SERCA2a.

Variables	Model (OPA1)				Model (SERCA2a)			
	*β*	95% CI	95% CI	*p* value	*β*	95% CI	95% CI	*p* value
		Lower	Upper			Lower	Upper	
Delta GLS	−0.066	−0.089	−0.044	<0.001	−0.029	−0.34	−0.178	<0.001
Delta GCS	−0.006	−0.033	0.021	0.639	−0.059	−0.156	0.046	0.271
Delta GRS	0.007	−0.026	0.04	0.654	−0.005	−0.130	0.120	0.993
Delta SDI	−0.034	−0.005	−0.012	0.004	−0.138	−0.217	−0.059	0.001
Delta LVEF	0.017	0.008	0.025	<0.001	0.074	0.046	0.103	<0.001

LVEF: ejection fraction left ventricular; GLS: global longitudinal strain; GRS: global radial strain; GCS: global circumferential strain; SDI: standard deviation index of three-dimensional.

**Table 4:** Multivariate linear regression analysis for the changes in mRNA levels of OPA1 and SERCA2a.

Variables	Model (OPA1)				Model (SERCA2a)			
	Adjusted *R*-square = 0.511				Adjusted *R*-square = 0.538			
	*β*	95% CI	95% CI	*p* value	*β*	95% CI	95% CI	*p* value
		Lower	Upper			Lower	Upper	
Delta GLS	−0.090	−0.145	−0.036	0.002	−0.229	−0.427	−0.030	0.026
Delta GCS	0.010	−0.010	0.031	0.317	0.003	−0.072	0.790	0.930
Delta GRS	−0.002	−0.027	0.023	0.895	−0.038	−0.130	0.053	0.393
Delta SDI	−0.005	−0.033	0.023	0.724	−0.020	−0.123	0.083	0.692
Delta LVEF	−0.009	−0.026	0.008	0.283	0.006	−0.056	0.068	0.848

LVEF: ejection fraction left ventricular; GLS: global longitudinal strain; GRS: global radial strain; GCS: global circumferential strain; SDI: standard deviation index of three-dimensional.
