# Supplementary material for: Early Right Ventricular Apical Pacing-Induced Gene Expression Alterations Are Associated with Deterioration of Left Ventricular Systolic Function
Source: Dis Markers. 2017 Aug 8;2017:8405196. doi: 10.1155/2017/8405196 (PMC5591927; doi:10.1155/2017/8405196)
Supplement: Supplementary file 1 — Table S1. Primer sequences used for quantitative real-time PCR. Table S2. Intra- and inter-observer variability of LVEF and strain parameters. [file 8405196.f1.pdf]

S1

Table 1. Primer sequences used for quantitative real-time PCR.

Gene	Primers	Sequences
**GAPDH**	Forward Primer	5’-CGGAGTCAACGGATTTGGTCGTAT-3’
	Reverse Primer	5’-AGCCTTCTCCATGGTGGTGAAGAC-3’
**OPA1**	Forward Primer	5’-GTGCTGCCCGCCTAGAAA-3’
	Reverse Primer	5’-TGACAGGCACCCGTACTCAGT-3’
**SCREA2a**	Forward Primer	5’-T GTGTAACGCCCTCAACAGC-3’
	Reverse Primer	5’-GAGAATCACGGGCAAGGAGAT-3’

S2

Table 2. Intra- and inter-observer variability of LVEF and strain parameters.

Variables	Intra-observer CV (%)	Inter-observer CV (%)
**LVEF**	5.8	7.1
**GLS**	7.3	8.7
**GRS**	11.2	12.8
**GCS**	13.6	15.2
**SDI**	12.5	13.6

LVEF, ejection fraction left ventricular; GLS, global longitudinal strain; GRS, global radial strain; GCS, global circumferential strain; SDI, standard deviation index of three-dimensional strain;CV, coefficient of variation.
